# Structural and electronic features of binary Li_2_S-P_2_S_5_ glasses

**DOI:** 10.1038/srep21302

**Published:** 2016-02-19

**Authors:** Koji Ohara, Akio Mitsui, Masahiro Mori, Yohei Onodera, Shinya Shiotani, Yukinori Koyama, Yuki Orikasa, Miwa Murakami, Keiji Shimoda, Kazuhiro Mori, Toshiharu Fukunaga, Hajime Arai, Yoshiharu Uchimoto, Zempachi Ogumi

**Affiliations:** 1 Office of Society-Academia Collaboration for Innovation, Kyoto University, Gokasho, Uji, Kyoto 611-0011, Japan; 2 Material Analysis Department, Material Development Division, TOYOTA MOTOR CORPORATION, 1, Toyota-cho, Toyota, Aichi 471-8572, Japan; 3 Research Reactor Institute, Kyoto University, 2-1010 Asashiro-Nishi, Kumatori-cho, Sennan-gun, Osaka 590-0494, Japan; 4 Graduate School of Human and Environmental Studies, Kyoto University, Yoshida-nihonmatsu-cho, Sakyo-ku, Kyoto 606-8501, Japan

## Abstract

The atomic and electronic structures of binary Li_2_S-P_2_S_5_ glasses used as solid electrolytes are modeled by a combination of density functional theory (DFT) and reverse Monte Carlo (RMC) simulation using synchrotron X-ray diffraction, neutron diffraction, and Raman spectroscopy data. The ratio of PS_*x*_ polyhedral anions based on the Raman spectroscopic results is reflected in the glassy structures of the 67Li_2_S-33P_2_S_5_, 70Li_2_S-30P_2_S_5_, and 75Li_2_S-25P_2_S_5_ glasses, and the plausible structures represent the lithium ion distributions around them. It is found that the edge sharing between PS_*x*_ and LiS_y_ polyhedra increases at a high Li_2_S content, and the free volume around PS_*x*_ polyhedra decreases. It is conjectured that Li^+^ ions around the face of PS_*x*_ polyhedra are clearly affected by the polarization of anions. The electronic structure of the DFT/RMC model suggests that the electron transfer between the P ion and the bridging sulfur (BS) ion weakens the positive charge of the P ion in the P_2_S_7_ anions. The P_2_S_7_ anions of the weak electrostatic repulsion would causes it to more strongly attract Li^+^ ions than the PS_4_ and P_2_S_6_ anions, and suppress the lithium ionic conduction. Thus, the control of the edge sharing between PS_*x*_ and LiS_y_ polyhedra without the electron transfer between the P ion and the BS ion is expected to facilitate lithium ionic conduction in the above solid electrolytes.

Lithium ion batteries (LIBs) have been widely used in many applications such as mobile phones, electric vehicles (EVs), and plug-in hybrid electric vehicles (PHVs). To improve their performance in such applications, solid electrolytes have attracted attentions^1^,^2^,^3^, because the realization of an all solid-state battery will enable the miniaturization of battery packages and increase the safety of battery components, compared with that of LIBs with an organic electrolyte. Binary Li_2_S-P_2_S_5_ glasses, which consist of PS_*x*_ polyhedral anions, are well-known superionic conductors, and are also candidates for such solid electrolytes owing to their high ionic conductivity at room temperature^4^,^5^. Recently, novel solid lithium electrolytes such as 70Li_2_S-29P_2_S_5_-1P_2_S_3_^6^, 75Li_2_S-25P_2_S_5_-50Li_2_S-25GeS_2_ (Li_10_GeP_2_S_12_)^7^,^8^, and 75Li_2_S-25P_2_S_5_-200Li_2_S-100SiS_2_ (Li_11_Si_2_PS_12_)^9^ have been reported, which feature liquidlike Li^+^ ion conduction. All these materials are derived from Li_2_S-P_2_S_5_ glasses. Therefore, the nature of Li_2_S-P_2_S_5_ glasses must be classified in detail to continue the development of advanced Li ionic conductors targeted at the realization of all-solid-state batteries. To clarify the origin of the ionic conduction, the structures of Li_2_S-P_2_S_5_ glasses have been thoroughly explored^10^,^11^,^12^ by reverse Monte Carlo (RMC) simulation^13^,^14^. A structural analysis of 70Li_2_S-30P_2_S_5_ glass by RMC simulation based on X-ray and neutron diffraction suggests that a large number of vacancies, which are recognized as fully acceptable units of a Li^+^ ion, are found around the PS_4_ tetrahedral anion^10^ and this structural feature is associated with high Li ionic conductivity^15^,^16^. Furthermore, the conduction pathways of the Li^+^ ions for 50Li_2_S-50P_2_S_5_, 60Li_2_S-40P_2_S_5_, and 70Li_2_S-30P_2_S_5_ glasses were also determined using a combination of RMC simulation and the bond valence sum method^11^, which suggests that the activation energy of Li^+^ ion conduction depends on the conduction pathway. However, structural models based on RMC simulation usually do not take the electronic structure into account, which could lead to incorrect electronically conductive structures for solid electrolyte materials that should be insulators.

In this paper, we present a comparative fundamental study of the structures of the 67Li_2_S-33P_2_S_5_ (67Li_2_S), 70Li_2_S-30P_2_S_5_ (70Li_2_S), and 75Li_2_S-25P_2_S_5_ (75Li_2_S) glasses. The lithium ionic conductivities of 67Li_2_S, 70Li_2_S, and 75Li_2_S were 5.6 × 10^−5^ S/cm, 1.4 × 10^−4^ S/cm, and 3.0 × 10^−4^ S/cm, respectively. We also analyze the environment of the Li^+^ ions on the basis of structural analyses combining X-ray and neutron diffraction with the aid of density functional theory (DFT)/RMC simulation and Raman spectroscopy to reveal the relationship between structural properties and Li ionic conduction.

## Results and Discussion

**Figure 1:**
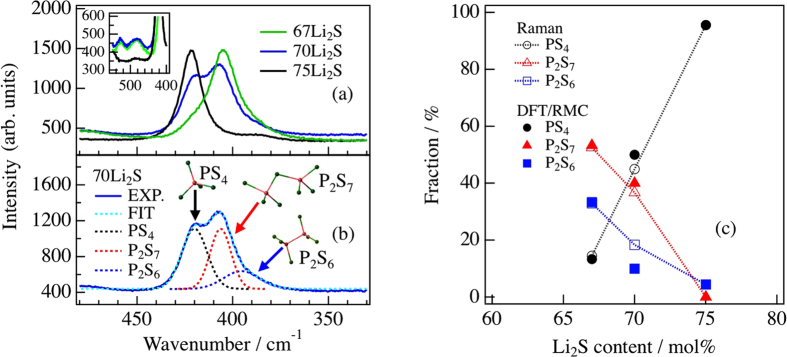
(**a**) Raman spectra in the range of 330–480 cm^−1^ for Li_2_S-P_2_S_5_ glasses. Black, blue, and green lines represent 75Li_2_S, 70Li_2_S, and 67Li_2_S glasses, respectively. The spectra in the range of 400–560 cm^−1^ are enlarged in the inset for clarity. (**b**) Spectral decomposition of Raman spectrum for 70Li_2_S glass. Blue line, experimental data; dotted lines, the fitting result for all PS polyhedra (right-blue), PS_4_ (black), P_2_S_7_ (red), and P_2_S_6_ (blue) anions. (**c**) PS_*x*_ polyhedral fractions for Li_2_S-P_2_S_5_ glasses derived from Raman spectra (open marks) and DFT/RMC model (filled marks).

To quantitatively evaluate the fraction of PS_*x*_ polyhedral anions, the Raman spectra of the 67Li_2_S, 70Li_2_S, and 75Li_2_S glasses were obtained, as shown in Fig. 1a. It is known that bands in the frequency range of 330–480 cm^−1^ are sensitive to the S-P-S bond angle. On the basis of previous studies^4^,^17^, we assigned the three bands at approximately 425 cm^−1^, 410 cm^−1^, and 390 cm^−1^ to the stretching vibration of the P-S bonds in the 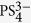 (ortho-thiophosphate) ion, 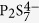 (pyro-thiophosphate) ion, and 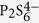 (an ethanelike structure with a P-P bond) ion, respectively. Since the scattering coefficient of the Raman spectroscopy is affected by each PS_*x*_ polyhedral anion^18^,^19^, the ratios of the 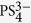, 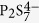, and 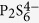 ions were estimated by a Lorentzian function, shown as dotted lines in Fig. 1b, and are summarized as open circles, open triangles, and open squares in Fig. 1c, respectively. It is clear that the ratio of 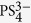 ions increases with the Li_2_S content, while the ratios of 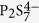 and 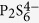 ions decrease. This tendency is in good agreement with that observed in previous studies^4^,^20^. Furthermore, the band corresponding to the stretching vibration of the P-P bond also disappears at approximately 530 cm^−1^ in the 75Li_2_S glass, as shown in the inset of Fig. 1a. Since the band at 547 cm^−1^ for polycrystalline Li_4_P_2_S_6_ is characterized by the stretching vibration of the P-P bonds^21^, the bands observed at 530 cm^−1^ in the glasses are related to the P-P stretching vibration. The disappearance of this band is consistent with the decrease in the ratio of 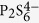 ions. Intriguingly, it was found that 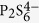 ions exist in these glasses with ratios of approximately 33.0%, 18.3% and 4.4% in 67Li_2_S, 70Li_2_S, and 75Li_2_S, respectively, whereas they should not be contained in the stoichiometric compositions (0PS_4_:100P_2_S_7_ in 67Li_2_S, 50PS_4_:50P_2_S_7_ in 70Li_2_S, and 100PS_4_:0P_2_S_7_ in 75Li_2_S). This means that there is a sulfur deficiency in these glasses. The sulfur deficiency was confirmed by an Inductively Coupled Plasma (ICP) analysis, as shown in Fig. S1. Hayashi *et al.* found by NMR measurement^22^ that a small number of 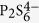 ions are formed in the glass ceramic 70Li_2_S-28P_2_S_5_-2P_2_S_3_, and the degradation of conductivity is expected to be caused by the formation of 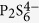 ions.

**Figure 2:**
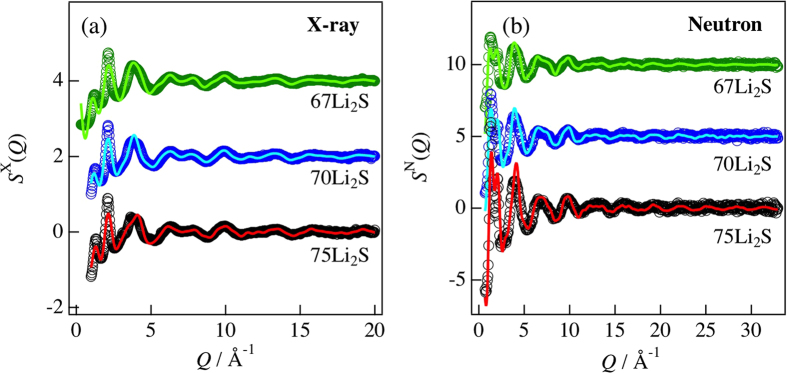
Total structure factors *S*(*Q*) at room temperature for Li_2_S-P_2_S_5_ glasses derived from (**a**) X-ray and (**b**) neutron diffraction. Circles, experimental data; lines, DFT/RMC model. The circle and line colors correspond to those in Fig. 1.

Figure 2a,b show experimental X-ray and neutron total structure factors, *S*^X^(*Q*) and *S*^N^(*Q*), respectively, for the 67Li_2_S, 70Li_2_S, and 75Li_2_S glasses. Oscillations in both *S*^X^(*Q*) and *S*^N^(*Q*) remain up to the high *Q* region, which is evidence for well-defined short-range order in the formation of P-S bonds. The difference between the three compositions is not significant in both sets of diffraction data, but the contrast between the diffraction data for *S*^X^(*Q*) and *S*^N^(*Q*) is apparent in the low-*Q* region. To extract quantitative information from the diffraction regarding the atomic arrangements in the glassy materials, the total pair distribution functions, *T*(*r*), were calculated for each glass by Fourier transformation of the total structure factor, *S*^X,N^(*Q*). Figure S2 shows *T*(*r*) for 75Li_2_S glass. From the negative coherent scattering length of neutrons for ^7^Li (hereafter the superscript is omitted), it is possible to identify the P-S and Li-S correlation lengths. The first peak at approximately 2.0 Å in both sets of diffraction data is related to the P-S correlation associated with the PS_4_ tetrahedral anions, while the second negative peak at approximately 2.5 Å is related to the Li-S correlation length. Both of these lengths are similar for all compositions and hence do not provide any specific information to help identify the structural features.

To uncover the relationship between the glassy structure and the high ionic conductivity for these glasses^4^,^7^,^9^,^23^, we modeled the atomic structure of the Li_2_S-P_2_S_5_ glasses by DFT/RMC simulation using X-ray and neutron diffraction data, fixing the ratios of the 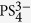, 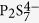, and 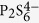 ions on the basis of Raman spectroscopy measurements to reproduce the plausible glassy structures. The total structure factors *S*^X,N^(*Q*) of the Li_2_S-P_2_S_5_ glasses derived from the DFT/RMC model are shown in Fig. 2 as lines. The DFT/RMC model is consistent with the experimental data. The peak observed at approximately *Q* = 1.2–1.3 Å^−1^ is usually called the first sharp diffraction peak (FSDP) and is thought to be a signature of the network structure formed in glassy materials. In phosphate glasses the FSDP is principally found approximately *Q* = 1.3 Å^−1 ^24, suggesting an intermediate range order of ~4.8 Å. This scale is larger than length between the centers of bonded PO_4_ tetrahedral anions (P-P correlation length, ~2.9 Å). This is related to the formation of a strong network mainly comprising corner-shared interconnections of regular PO_4_ tetrahedral anions (sp^3^ bonding)^25^. The GeS_2_-P_2_S_5_ glasses also have an FSDP at approximately *Q* = 1.1–1.2 Å^−1^, suggesting intermediate range order between GeS_4_ or PS_4_ tetrahedral anions^26^. On the other hand, the FSDP in the Li_2_S-P_2_S_5_ glasses was observed at approximately *Q* = 1.2 Å^−1^, corresponding to an intermediate range order of ~5.0 Å. However, note that the corner-shared interconnections of the regular PS_4_ tetrahedral anions are not related to the FSDP in this system. Because we fixed the ratios of the P_*x*_S_*y*_ tetrahedral anions and the interconnections of the regular PS_4_ tetrahedral anions in the DFT/RMC simulations. To understand the origin of the FSDP, the partial structure factors for the 70Li_2_S glass were calculated from the DFT/RMC model as shown in Fig. S3. As can be seen in this figure, *S*_P−P_(*Q*) has an FSDP at approximately *Q* = 1.2 Å^−1^, indicating that the P-P correlation contributes to the formation of the FSDP. According to a previous study^10^, the X-ray weighting factor for the P-P Faber-Ziman partial structure factor is 0.0346 (as evaluated from the form factor values at *Q* = 0), whereas the corresponding neutron weighting factor is 0.2438 for the 70Li_2_S glass. Actually, the heights of the FSDP in *S*^N^(*Q*) are greater than those in *S*^X^(*Q*). Not only glasses, but also the molecular liquids CCl_4_, SiCl_4_, GeCl_4_, and SnCl_4_ with the regular *X*Cl_4_ tetrahedral anion (*X* = C, Si, Ge, and Sn) have a similar FSDP at approximately *Q* = 1.2 Å^−1^, despite these liquids not having the interconnections of regular *X*Cl_4_ tetrahedral anions^27^. Although the relationship between the FSDP and the topology of the network is still not well understood, it is suggested that a pseudo network of P-P correlations contributes to the stabilization of the glassy structure.

To obtain information on the partial correlation in real space, the partial pair distribution functions (PDFs), *g*_ij_(*r*), for the Li_2_S-P_2_S_5_ glasses were calculated from the DFT/RMC model as shown in Fig. S4. *g*_ij_(*r*) for P-S correlation, *g*_P−S_(*r*), has a peak at approximately *r* = 2.0 Å and a shoulder on the high-*r* side. This bond length is consistent with that of a bridging sulfur (BS) in a P-S-P bond. *g*_Li−S_(*r*) also has a peak at approximately *r* = 2.5 Å, which is consistent with the experimental results for the PDF. Furthermore, *g*_P−P_(*r*) has two peaks at *r* = 2.2 Å and *r* = 3.5 Å, corresponding to the bond length of the 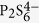 ion and the correlation length of the 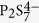 ion, respectively. All the *g*_ij_(*r*) peaks except for Li-Li correlation are well defined and sharp because the combination of X-ray diffraction, neutron diffraction, and DFT calculation provides us with a sufficient number of factors for each correlation. Note that the difference between the three glasses is very small, suggesting that their atomic correlations are very similar. This behavior is consistent with the structure factors, *S*^X,N^(*Q*), obtained from the DFT/RMC simulations, where the data are similar for the three glasses.

**Figure 3:**
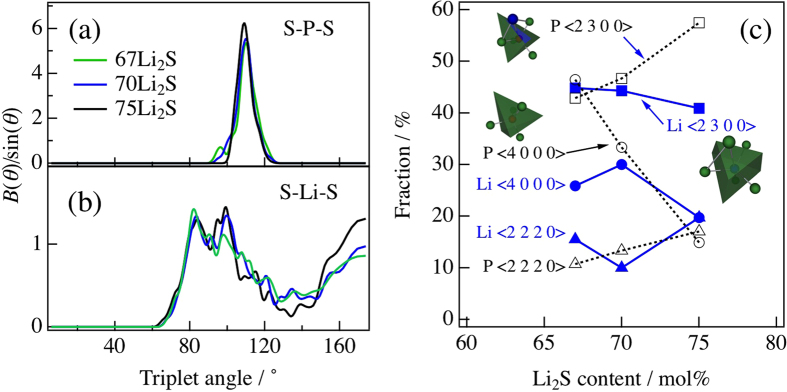
Bond angle distributions for (**a**) S-P-S and (**b**) S-Li-S derived from DFT/RMC model. Line colors correspond to those in Fig. 1. (**c**) Comparison between the P-centered (open marks with dotted line) and Li-centered Voronoi polyhedra (filled marks with solid line) for Li_2_S-P_2_S_5_ glasses derived from DFT/RMC model.

To understand the short-range correlation in detail, the coordination numbers in the Li_2_S-P_2_S_5_ glasses calculated up to 3.2 Å are summarized in Table 1. The coordination number of S around P, *N*_P−S_, increases with increasing Li_2_S content owing to the disappearance of the 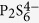 ions. *N*_P−Li_ also increases, while *N*_Li−P_ and *N*_Li−S_ remains almost constant in the three compositions. Furthermore, as the simplest analysis beyond two-body correlations, the bond angle distributions of the S-P-S and S-Li-S triplets for the 67Li_2_S, 70Li_2_S and 75Li_2_S glasses were calculated from the DFT/RMC model as shown in Fig. 3a,b, respectively. The S-P-S has a peak at 109° for all compositions owing to the formation of the regular PS_4_ tetrahedral anion with *sp*^3^ bonding. It also has a peak at approximately 95° except for the 75Li_2_S glass, which is related to the existence of BS ions. The S-Li-S bond angle distribution also shows a similar trend for all compositions regardless the BS ions of the existence, but the magnitude of the peak at approximately 100° significantly increases at a higher Li_2_S content. This increase is closely related to the change in the coordination environment of the Li^+^ ions.

To obtain the coordination environment of the Li^+^ ions in detail, Voronoi polyhedron statistics were calculated by Voronoi tessellation analysis^28^,^29^, in which it is assigned by a Voronoi index 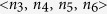, where 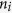 denotes the number of *i*-edged faces and 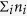 is the total coordination number. The results for Li-centered Voronoi polyhedra calculated up to 3.2 Å are shown in Fig. 3c, together with results for P-centered polyhedra. This calculation length corresponds to the first coordination length of the Li-S correlation determined by the DFT/RMC model. Therefore, the calculation of P-centered Voronoi polyhedra includes information about the coordination environment beyond the first coordination of P-S correlation. The P-centered Voronoi polyhedra up to the first coordination environment have no composition dependence as shown in Fig. S5. It is clear that the fractions of <2 3 0 0> and <2 2 2 0> P-centered Voronoi polyhedra beyond the first coordination environment increase relative to that of <4 0 0 0> Voronoi polyhedra in the Li_2_S-P_2_S_5_ glasses, as shown by black dotted lines in Fig. 3c, which is consistent with the coordination number for P-Li correlation, *N*_P−Li_. This increase in the fraction of higher-index Voronoi polyhedra occurs with increasing Li_2_S content, which indicates that the number of Li^+^ ions increases at around the PS_*x*_ polyhedral anion. On the other hand, the Li-centered Voronoi polyhedra are shown in Fig. 3c as blue solid lines and have no dependence on the Li_2_S content, which is also consistent with the coordination number for Li-P correlation, *N*_Li−P_. Although the distribution of Li^+^ ions has not been characterized, it has been found that the simple Voronoi polyhedra for Li-centered polyhedra are dominant in the DFT/RMC model owing to the consideration of the electron state for Li^+^ ions in the DFT calculation. It is suggested that the free volume around PS_*x*_ polyhedral anions allows the distribution of Li^+^ ions at a higher Li_2_S content.

**Figure 4:**
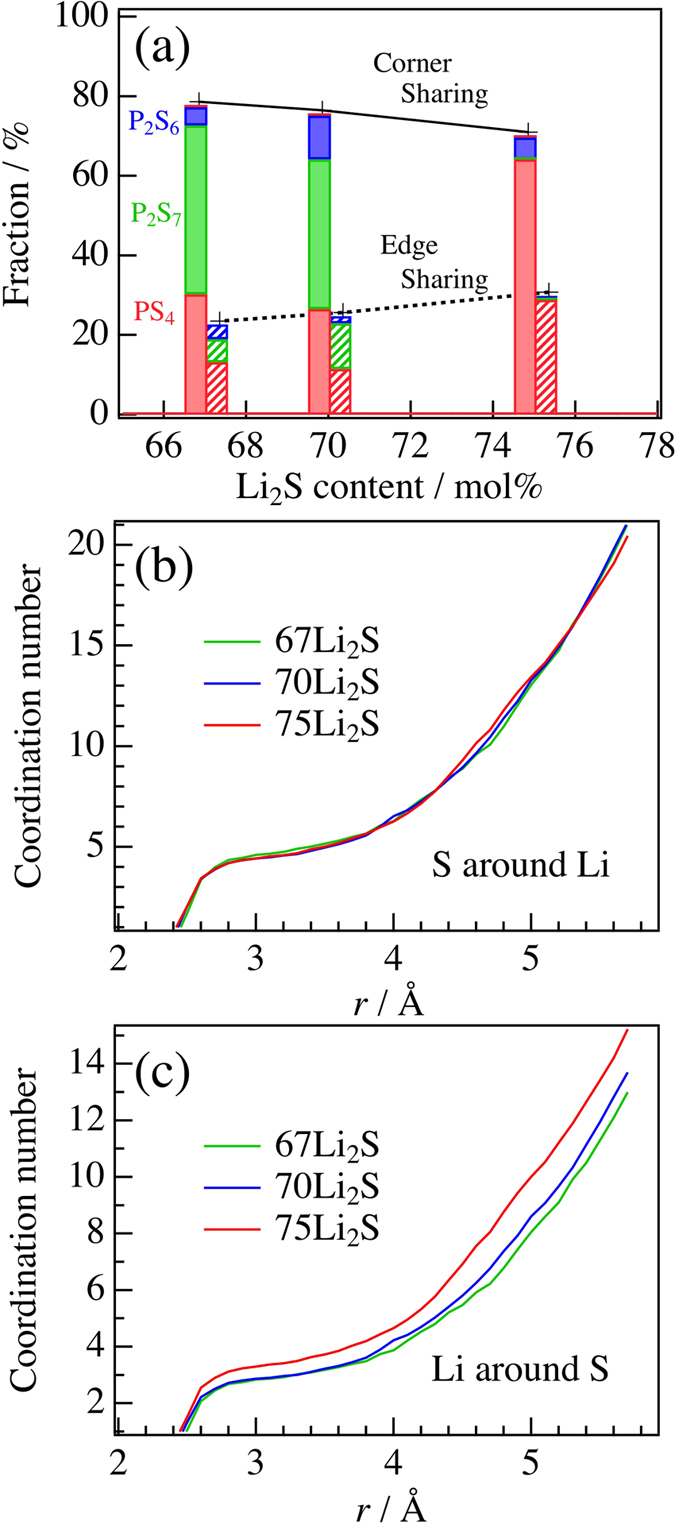
(**a**) Polyhedral connection statistics for Li_2_S-P_2_S_5_ glasses calculated using DFT/RMC model. All connections are between PS_*x*_ and LiS_*y*_ polyhedra. Red, green, and blue represent 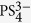, 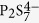, and 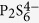 ions, respectively. Filled and hatched bars represent corner and edge sharing. Relationship between the bond length and the coordination number for the Li-S (**b**) and S-Li (**c**) correlations derived using the DFT/RMC model.

**Figure 5:**
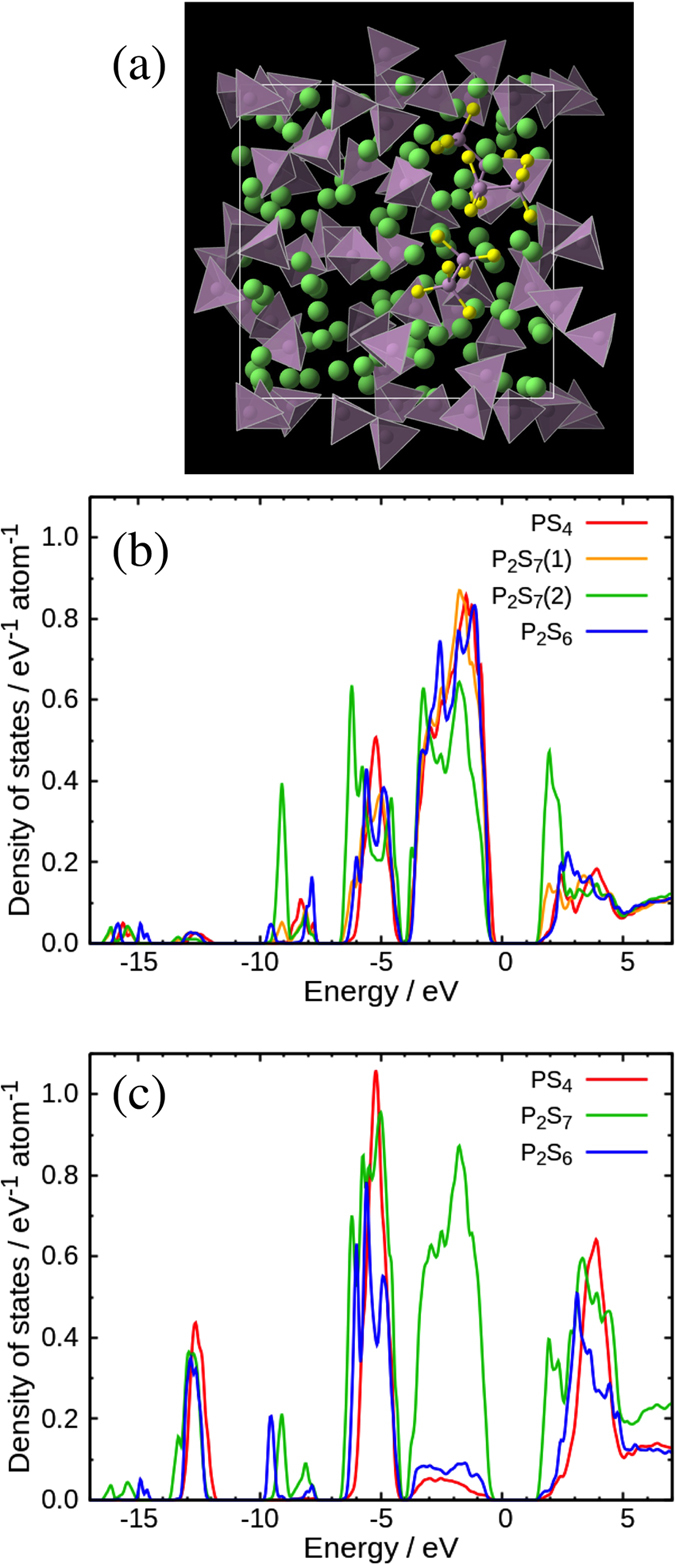
(**a**) DFT/RMC model of 70Li_2_S glass. Green, Li; Purple, P and PS polyhedral anions; Yellow, S. Polyhedral partial DOS for (**b**) S *p*-orbital and (**c**) P *p*-orbital of 70Li_2_S glass.

The extent of polyhedral connection between PS_*x*_ and LiS_*y*_ polyhedra in the Li_2_S-P_2_S_5_ glasses through corner, edge, and face sharing is calculated as shown in Fig. 4a. The results were classified on the basis of PS_*x*_ polyhedral anions. The filled and hatched bars indicate corner and edge sharing, respectively. The fraction of edge sharing increases relative to that of corner sharing with increasing Li_2_S content, which is related to the bond angle distribution of the S-Li-S triplet. This means that an increase of the Li_2_S content does not affect the local coordination environment of Li^+^ ions, and free volume around the PS_*x*_ polyhedral anion decreases. On the other hand, each fraction of the PS_*x*_ polyhedral anions in the 67Li_2_S and 70Li_2_S glasses is almost the same, although we expected a distinct difference between the compositions. This result shows the distribution of Li^+^ ions in each PS_*x*_ polyhedral anion. Strangely, the ratio (the number of Li ions sharing S with P_2_S_7_ anions/the number of Li ions sharing S with PS_4_ anions) in 70Li_2_S was found to be about 1.28, which is larger than the value of 1.11 for 67Li_2_S. The ratios of the molecular anions (P_2_S_7_/PS_4_) are 0.82 and 3.62 for 70Li_2_S and 67Li_2_S, respectively. This suggests that the P_2_S_7_ anion attracts the Li^+^ ions more than the PS_4_ and P_2_S_6_ anions. To evaluate the free volume, we calculated the bond length to the coordination number of the S around Li, *N*_Li−S_, and Li around S, *N*_S−Li_, as shown in Fig. 4b,c. The maximum bond length of 5.7 Å was determined from the Li-S-P bond length (3.2 Å + 2.5 Å), corresponding to the calculation length of polyhedral connection statistics. As can be seen in Fig. 4b, the *N*_Li−S_ in this system has no composition dependence, which is consistent with the Li-centered Voronoi polyhedra. On the other hand, the *N*_S−Li_ in 75Li_2_S is found to be larger than that of others as shown in Fig. 4c, which suggests that the free volume around S ions decreases in 75Li_2_S. Intriguingly, the *N*_S−Li_ increase in 70Li_2_S compared to that in 67 Li_2_S at 3.8 Å and over. Thus, as the Li_2_S content increases in this system, the free volume around Li ion has no composition dependence, while that around S decreases. The DFT/RMC structure is consistent with both the diffraction data and the Raman data (Fig. 5a), and we compared the electronic structure in terms of each PS_*x*_ polyhedral anion for the 70Li_2_S glass. Figure 5b,c show the partial density of states (p-DOS) of the 70Li_2_S glass for the S 3*p*-orbital and P 3*p*-orbital, respectively. It is apparent that the orbitals form a hybrid orbital between the phosphorus and sulfur; the highest occupied molecular orbital (HOMO) is located at −4.0–−0.5 eV and the lowest unoccupied molecular orbital (LUMO) is located at 1.5–5.0 eV. The positive charge of the P ion is large owing to the hybrid orbital. However, the p-DOS plots of P ion reveal that the P_2_S_7_ anion only differs from the PS_4_ and P_2_S_6_ anions (Fig. 5c). A shallow level appears near the bottom of the LUMO at approximately 2.0 eV in the P_2_S_7_ anion, which relates to a covalent bond between the P ion and the BS ion in the P_2_S_7_ anion. This electron transfer is expected to weaken the positive charge of the P ions, which attract Li^+^ ions to the P_2_S_7_ anions more strongly than the other PS_*x*_ polyhedral anions. Furthermore, the attracted Li^+^ ions are easy to stay around the P_2_S_7_ anions, which may suppress the lithium ionic conduction in solid electrolytes. On the other hand, the P_2_S_6_ anion is almost the same to the PS_4_ anion in terms of the electronic structure and does not suppress the Li ion conduction compared to the P_2_S_7_ anion, although we expected a strong suppression to Li ion as shown Hayashi *et al.*^22^. It is well known that the diffusion of cations is accelerated by the polarization of anions^30^,^31^,^32^. Also, the Li^+^ ion distribution is clearly affected by the polarization of anions^33^, which means that the edge sharing between PS_*x*_ and LiS_*y*_ polyhdera is related to the lithium ionic conduction. Actually, the lithium ionic conductivity of 75Li_2_S without P_2_S_7_ anions is higher than that of the other glasses. Furthermore, Li_2_S-SiS_2_ glasses with added LiI with exhibiting large polarization have a high ionic conductivity^34^. Thus, the control of the edge sharing between PS_*x*_ and LiS_*y*_ polyhdera without the electron transfer between the P ion and the BS ion is expected to facilitate the lithium ionic conduction in a solid electrolyte, which should contribute to the development of all-solid batteries.

## Conclusion

In this study, we found that 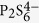 ions as well as 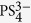 and 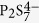 ions are present in 67Li_2_S-33P_2_S_5_ (67Li_2_S), 70Li_2_S-30P_2_S_5_ (70Li_2_S), and 75Li_2_S-25P_2_S_5_ (75Li_2_S) glasses on the basis of Raman spectroscopy measurement. Density functional theory and reverse Monte Carlo simulations (DFT/RMC) quantitatively reproduced the results of high-energy X-ray diffraction, neutron diffraction, and Raman spectroscopy, fixing the ratios of 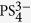, 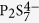, and 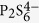 ions. The DFT/RMC model indicates that the P-P correlation contributes to the formation of the first sharp diffraction peak, suggesting that the structure can be stabilized by this correlation in the three glasses. The distinct peak at approximately 100° for the S-Li-S bond angle distribution at a high Li_2_S content is consistent with the increase in the edge sharing polyhedral connection between PS_*x*_ and LiS_*y*_, which means that the free volume around the PS_*x*_ polyhedral anion allows the distribution of Li^+^ ions. It is conjectured that Li^+^ ions around the face of the PS_*x*_ polyhedra are affected by the polarization of anions. The electronic structure of the DFT/RMC model suggests that the existence of the P_2_S_7_ anion may suppress lithium ionic conduction. Thus, it has been demonstrated that the observation of the local structure is important for understanding the origin of high lithium ionic conduction. We suggest that the high ionic conduction in solid electrolytes can be controlled by the edge sharing between PS_*x*_ and LiS_*y*_ polyhedra without the electron transfer between the P ion and the BS ion. This finding is a crucial key concept for designing new solid electrolytes.

## Methods

### Sample preparation

The Li_2_S-P_2_S_5_ glasses were prepared by the mechanical milling method. ^7^Li_2_S (Kojundo Chemical lab., Ltd., 99.8%) and P_2_S_5_ (Aldrich, 99%) crystalline powders were used as the starting materials. A mixture of these materials was mechanically milled at room temperature by a planetary ball mill using a zirconia pot (45 ml) with 10 zirconia balls (diameter: 10 mm). The rotation speed was 370 rpm and the milling time was about 80 h. All the processes were performed in a dry Ar atmosphere. Ionic conductivity was measured by the AC impedance method in an Ar atmosphere at room temperature with an applied frequency range of 100 Hz to 1 MHz using a Solartron 1260 frequency response analyzer. Carbon-coated blocking the electrode was painted on both sides of the sample. The observed ionic conductivities of 67Li_2_S, 70Li_2_S, and 75Li_2_S were 5.6 × 10^−5 ^S/cm, 1.4 × 10^−4 ^S/cm, and 3.0 × 10^−4 ^S/cm, respectively. Densities of Li_2_S-P_2_S_5_ glasses were measured using a gas pycnometer (Accupyc 1330, Micromeritics) under a high purity He atmosphere at room temperature.

### Raman spectroscopic measurement

Raman spectra for the Li_2_S-P_2_S_5_ glasses were acquired at room temperature on a LabRAM HR-800 (Horiba-Jobin Yvon) spectrometer equipped with a 100× lens (NA = 0.90, Olympus), a 1800 grooves/mm grating, and an excitation wavelength of 632.8 nm (He-Ne laser). The laser power was reduced to less than 1 mW to avoid laser-induced degradation on the focused particles (laser spot size; 4 *μ*m in diameter). The exposure time was 30 s × 10 times for several particles.

### High-energy X-ray diffraction measurement

The high-energy X-ray diffraction experiments for the Li_2_S-P_2_S_5_ glasses were carried out at room temperature at the SPring-8 high-energy XRD beamline BL04B2 using a two-axis diffractometer^35^. The incident X-ray energy obtained from a Si 220 crystal monochromator was 61.4 keV. The diffraction patterns of the samples and an empty tube were measured in the transmission geometry with an angle from 0.3 to 40°, corresponding to a *Q*-range from 0.2 to 20 Å^−1^. The intensity of the incident X-ray was monitored in an ionization chamber filled with Ar gas and the scattered X-rays were detected by a Ge detector. A vacuum electric chamber was used to suppress air scattering around the sample. The collected datasets were corrected for the absorption, background, and polarization effects. Details of the data correction and normalization procedures are given in ref. 35.

### Time-of-flight neutron diffraction measurement

The time-of-flight neutron diffraction experiments for the Li_2_S-P_2_S_5_ glasses were carried out at room temperature using the General Materials Diffractometer (GEM) at ISIS, Rutherford Appleton Laboratory, UK^36^,^37^. The data were reduced and corrected for attenuation and multiple scattering using the Gudrun program^38^.

### Density functional theory and Reverse Monte Carlo simulations

To obtain a plausible structural model, we evaluated the densities of the glasses (Fig. S6). DFT/RMC simulations of the Li_2_S-P_2_S_5_ glasses were carried out from an initial atomic configuration prepared by an Amorphous Cell code (BIOVIA) in a cubic box with the corresponding densities listed in Table 2. The ratios of the 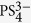, 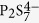, and 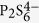 ions in the Li_2_S-P_2_S_5_ model structures were fixed on the basis of the Raman spectroscopic results. The numbers of Li^+^ ion was fixed to satisfy the stoichiometry of the PS_*x*_ polyhedral anions. DFT and RMC calculations were performed iteratively until the difference in the atomic coordination between DFT and RMC became less than 0.2 Å. DFT calculations were performed using the projector augmented wave (PAW) method^39^ implemented in VASP^40^,^41^. The generalized gradient approximation (GGA) functional of Perdew, Burke, and Ernzerhof (PBE)^42^ was used for the exchange correlation term. A plane-wave cutoff energy of 260 eV was used. Internal atomic positions were optimized until the residual forces became less than 5 × 10^−2 ^eV/Å. The experimental *S*(*Q*) obtained from the high-energy X-ray and neutron diffraction measurements were fitted simultaneously by employing the RMC++ code^43^. The cut off radius, *i.e.*, the minimum allowed distance between atom pairs, was estimated from the experimental pair distribution function. The simulations were performed for different initial configurations for each composition, and their validity was verified.

## Additional Information

**How to cite this article**: Ohara, K. *et al.* Structural and electronic features of binary Li_2_S-P_2_S_5_ glasses. *Sci. Rep.* **6**, 21302; doi: 10.1038/srep21302 (2016).

## Supplementary Material

Supplementary Information

## Figures and Tables

**Table 1:** Average coordination numbers in Li_2_S-P_2_S_5_ glasses up to *r* = 3.2 Å derived from the DFT/RMC model.

Samples	67Li_2_S glass	70Li_2_S glass	75Li_2_S glass
*N*_P−Li_	2.04	2.20	3.23
*N*_P−P_	0.36	0.13	0.09
*N*_P−S_	3.64	3.87	3.92
*N*_Li−Li_	0.55	0.66	0.79
*N*_Li−P_	0.98	0.94	1.11
*N*_Li−S_	4.74	4.57	4.58
*N*_S−Li_	2.93	2.96	3.41
*N*_S−P_	1.09	1.07	1.00
*N*_S−S_	0.40	0.28	0.21

*N*_i−j_: partial coordination number of *j* atoms around *i* atoms.

**Table 2:** Details of Li_2_S-P_2_S_5_ glasses; compositions, densities, atomic number densities, particle numbers, and box lengths used in DFT/RMC simulation.

Samples	67Li_2_S glass	70Li_2_S glass	75Li_2_S glass
Composition	(Li_2_S)_67_(P_2_S_5_)_33_	(Li_2_S)_70_(P_2_S_5_)_30_	(Li_2_S)_75_(P_2_S_5_)_25_
Density (g/cm^3^)	1.950	1.938	1.935
Atomic number density (Å^−1^)	0.0487	0.0496	0.0518
Particle number	180	208	368
Box length (Å)	15.39	16.11	18.35
