# Supplementary material for: Structural and electronic features of binary Li2S-P2S5 glasses
Source: Sci Rep. 2016 Feb 19;6:21302. doi: 10.1038/srep21302 (PMC4759574; doi:10.1038/srep21302)
Supplement: Supplementary Information [file srep21302-s1.pdf]

Supplementary Information for

Structural and electronic features of binary Li_2S-P_2S_5 glasses

Koji Ohara[1], Akio Mitsui[1,2], Masahiro Mori[1], Yohei Onodera[3], Shinya Shiotani[1], Yukinori Koyama[1], Yuki Orikasa[4], Miwa Murakami[1], Keiji Shimoda[1], Kazuhiro Mori[3], Toshiharu Fukunaga[3], Hajime Arai[1], Yoshiharu Uchimoto[4], and Zempachi Ogumi[1]

Affiliations

[1] Office of Society-Academia Collaboration for Innovation, Kyoto University, Gokasho, Uji, Kyoto 611-0011, Japan

[2] Material Analysis Department, Material Development Division, TOYOTA MOTOR CORPORATION, 1, Toyota-cho, Toyota, Aichi 471-8572, Japan

[3] Research Reactor Institute, Kyoto University, 2-1010 Asashiro-Nishi, Kumatori-cho, Sennan-gun, Osaka 590-0494, Japan

[4] Graduate School of Human and Environmental Studies, Kyoto University, Yoshida-nihonmatsu-cho, Sakyo-ku, Kyoto 606-8501, Japan

Figures

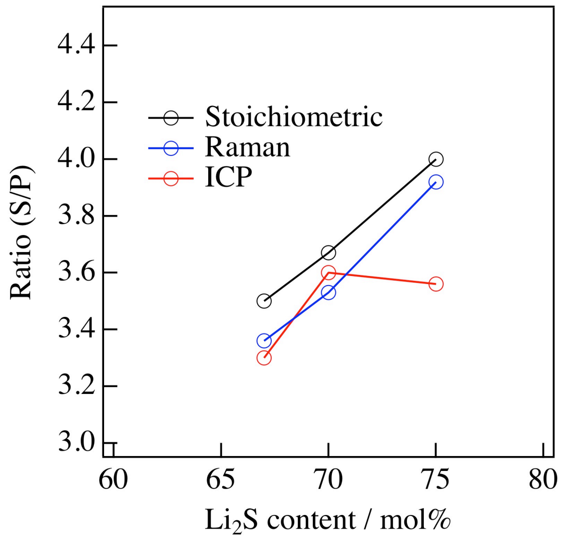

Figure S1 Ratio of sulfur to phosphorus for Li_2S-P_2S_5 glasses at room temperature determined using the stoichiometry, Raman spectra, and an Inductively Coupled Plasma (ICP) analysis, respectively.

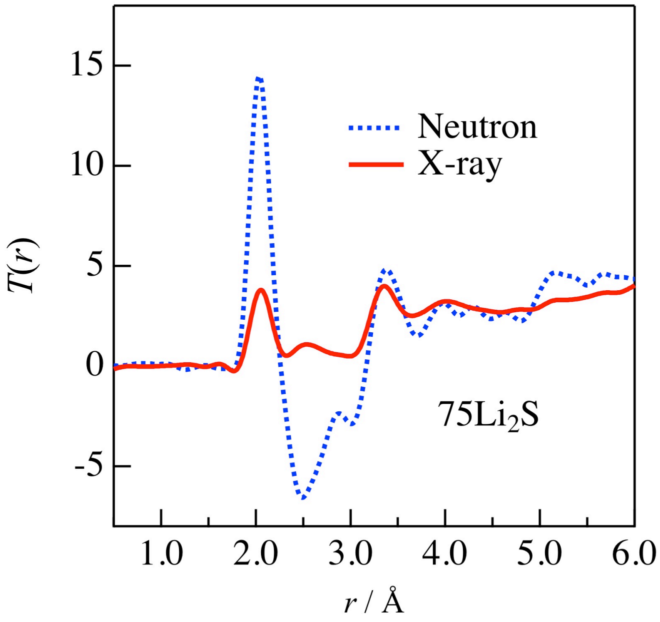

Figure S2 Total pair correlation functions $T(r)$ for $75Li_2S$ glasses derived from X-ray (red solid line) and neutron (blue dotted line) diffraction.

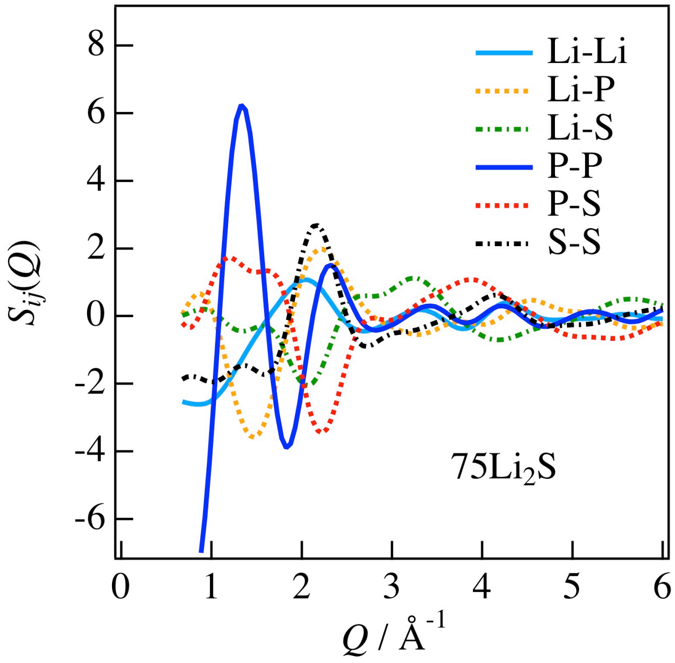

Figure S3 Partial structure factors $S(Q)$ for Li-Li (light blue solid line), Li-P (orange dotted line), Li-S (green dashed line), P-P (blue solid line), P-S (red dotted line), and S-S (black dashed line) derived from DFT/RMC model for $75Li_2S$ glass.

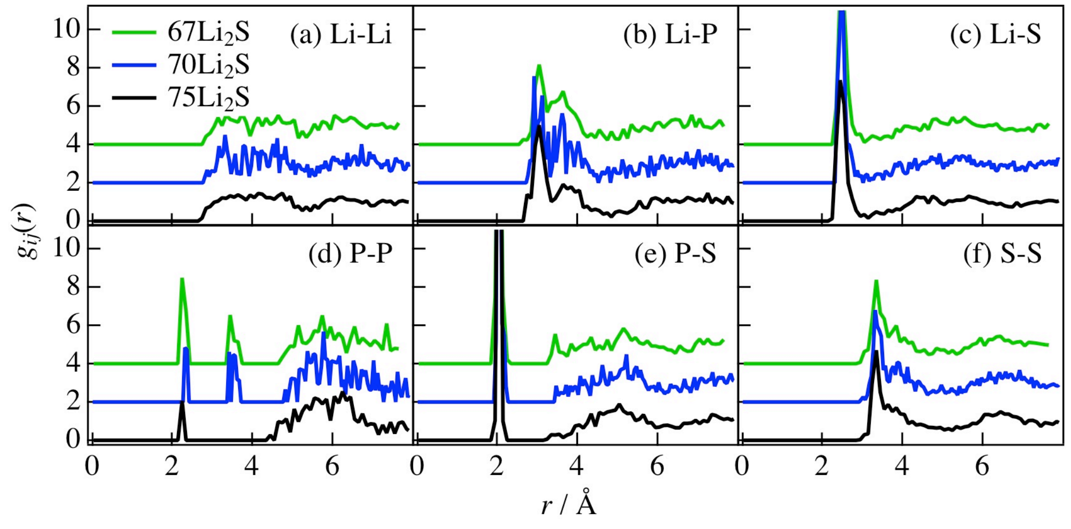

Figure S4 Partial pair distribution functions $g_{ij}(r)$ for Li-Li (a), Li-P (b), Li-S (c), P-P (d), P-S (e), and S-S (f) derived from DFT/RMC model for Li_2S-P_2S_5 glasses. Line colors correspond to those in Fig. 1.

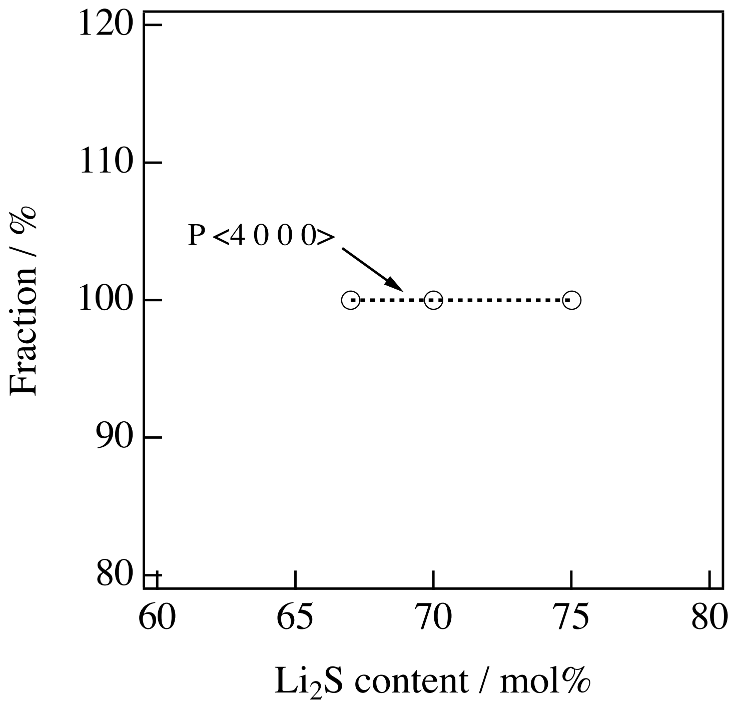

Figure S5 P-centered Voronoi polyhedra in the Li_2S-P_2S_5 glasses calculated up to 2.5 Å.

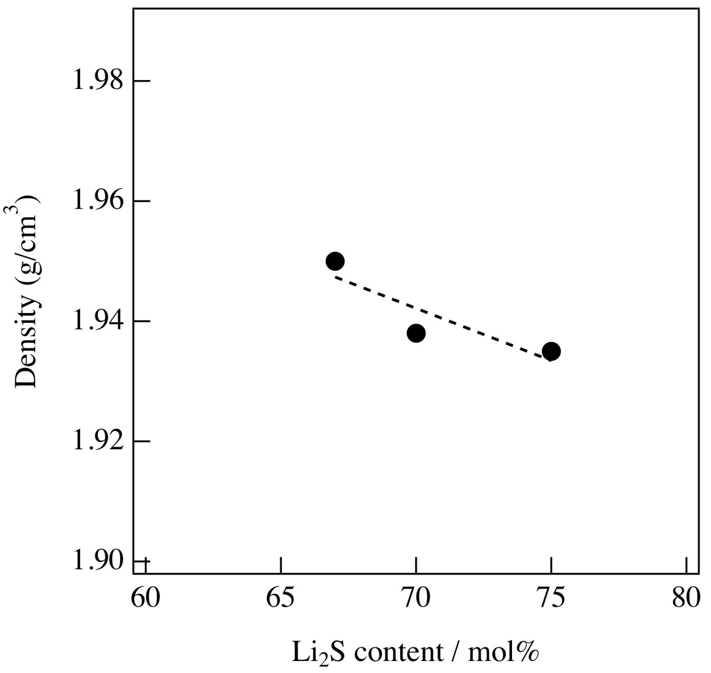

Figure S6 Densities of Li_2S-P_2S_5 glasses at room temperature determined using a pycnometer.

Table

Table S1. Distributions of Li- and P-centered Voronoi polyhedra for Li_2S-P_2S_5 glasses up to r = 3.2 Å derived from DFT/RMC model, which is assigned by a Voronoi index $<n_3, n_4, n_5, n_6>$, where n_i denotes the number of i-edged faces and $\Sigma_i n_i$ is the total coordination number.

Voronoi index	Coordination number	$67Li_2S$ (%)		$70Li_2S$ (%)		$75Li_2S$ (%)	
		Li-centered	P-centered	Li-centered	P-centered	Li-centered	P-centered
<4 0 0 0>	4	25.86	46.43	30.00	33.33	19.71	14.89
<2 3 0 0>	5	44.83	42.86	44.29	46.67	40.88	57.45
<0 6 0 0>	6	5.17	0	5.71	3.33	4.38	4.26
<2 2 2 0>	6	15.52	10.71	10.00	13.33	19.71	17.02
<1 3 3 0>	7	0	0	2.86	0	3.65	4.26
<2 2 2 1>	7	0	0	1.43	0	0	2.13
<0 5 2 0 >	7	1.72	0	1.43	0	1.46	0
<3 0 3 1>	7	0	0	1.43	0	0	0
